# Experimental and Numerical Investigation of Shielding Gas Behaviors in Laser Welding of TC4 Alloy

**DOI:** 10.3390/ma16196511

**Published:** 2023-09-30

**Authors:** Ao Chen, Bingchen Li, Xi Chen, Meng Jiang, Shuai Zou, Wang Tao, Zhenglong Lei, Yanbin Chen

**Affiliations:** 1 National Key Laboratory for Precision Hot Processing of Metals, Harbin Institute of Technology, Harbin 150001, China; chenao1993@126.com (A.C.); 23b909135@stu.hit.edu.cn (B.L.); 2 State Key Laboratory of Advanced Welding and Joining, Harbin Institute of Technology, Harbin 150001, China; 21s109289@stu.hit.edu.cn (S.Z.); taowang81@hit.edu.cn (W.T.); leizhenglong@hit.edu.cn (Z.L.); chenyb@hit.edu.cn (Y.C.)

**Keywords:** shielding gas, titanium alloy, laser welding, numerical simulation, protection effect

## Abstract

Gas protection is a crucial part of quality control in laser welding, especially for titanium alloy, which oxidizes easily at high temperatures. Substantial experiments concerning shielding gas characteristics in the welding process have been implemented. However, the common analysis conducted is simplistic and lacks a theoretical basis. This paper presented an investigation of the shielding gas behaviors based on numerical simulation and a titanium alloy laser welding experiment. The numerical model was established and validated by experiment. Subsequently, the temperature field and gas flow fields were calculated. By combining the two fields, the threshold temperature of gas protection was determined, and the influence of shielding gas parameters on the protection effect was examined. The results revealed that the protection of the high-temperature zone was primarily influenced by the nozzle height, nozzle inner diameter, and nozzle angle, while the plasma suppression effect was mainly correlated with the nozzle inner diameter and gas flow rate. These initial findings provide scientific guidance for the better quality production of laser beam welded components made of not only titanium alloy but also other metallic materials.

## 1. Introduction

On account of outstanding comprehensive properties, including high specific strength, prominent corrosion resistance, and great biocompatibility, titanium alloy has emerged as an excellent candidate in the fields of aerospace and biomedical science [1,2,3,4,5,6,7]. With the significant advantages of high energy density, high dimensional accuracy, and a high degree of automation, laser welding has received considerable attention and has been utilized more and more widely in industry [8,9,10,11,12,13,14,15,16]. Under high temperatures, titanium alloy strongly absorbs O_2_, N_2_, and H_2_. As the gas content increases, the gas atoms exist supersaturately in titanium, resulting in the formation of intermetallic compounds, which seriously deteriorate the comprehensive properties of titanium alloy [17,18]. When the protection effect of the high-temperature weld zone is favorable, the surface of the weld is a bright silver–white color. However, when the high-temperature weld zone protection effect worsens, more contamination enters the weld zone, which leads to a golden-yellow, blue, bluish-purple, and dark gray weld surface as the contamination content increases [19]. Furthermore, in the laser welding process of the titanium alloy, the plasma plume, which generates the shielding effect on the laser, occurs above the keyhole and causes a reduction in energy absorbed by the base metal and makes the welding process unstable [20].

Due to noticeable activeness at high temperatures, titanium alloy welds need sufficient protection during the laser welding process. Inert gas shielding is an extensively applied method to prevent the melting pool from being contaminated because of its convenience and cost-effectiveness. Moreover, shielding gas is able to suppress the vapor plume and enhance the laser energy absorption rate of base metal [21]. In recent years, there has been increasing interest in the study of shielding gas behaviors in laser welding. Gas flow rate is the most studied parameter owing to its convenience to be regulated. Blackburn et al. [22] revealed that better weld appearance could be acquired by a lower gas flow rate as a consequence of the turbulence caused by a high flow rate. Vyskoč et al. [23] investigated the influence of gas flow rate on the weld profile. A high gas flow rate could decrease the energy absorbed by plasma and enhance the weld width. T. et al. [24] discussed the correlation between the shielding gas blown distance and porosity. Optimum shielding gas blown distance was able to stabilize the keyhole and restrain the formation of pores in the weld. Apart from the gas flow rate, the gas nozzle posture was also investigated by some researchers. Campana et al. [25] found that the incidence angle of the shielding gas was preferably perpendicular to the surface of the workpiece. The nozzle height should be as low as possible in order to provide effective gas protection for the keyhole and the high-temperature weld zone. Wang et al. [26] studied the influence of nozzle inclination angle with the workpiece on the protection effect. The smaller the nozzle inclination angle, the larger the characteristic shield size of the assist gas will be. Lee et al. [27] implemented low vacuum laser welding of TC4 alloy and explored the effect of nozzle stand-off distance on the laser-induced plume generation and the degree of weld bead oxidation. When using argon as the shielding gas, a stand-off distance of 10 mm is necessary to prevent plume generation and weld oxidation.

From the aforementioned analysis, it could be seen that the results of various shielding gas behaviors studied by different investigators were not entirely consistent. The influences of shielding gas flow rate and gas nozzle posture on the protection of titanium alloy laser welding lack systematic research. Furthermore, the critical protection temperature of laser-welded titanium alloy has not been revealed.

In this work, finite element models of the temperature field and shielding gas flow field of titanium alloy laser welding were set up and validated by laser welding experiments. The inhomogeneity of the workpiece surface temperature was not taken into consideration, and both models were not coupled. Based on the finite element model, a threshold protection temperature of titanium alloy laser welding was derived. The patterns of shielding gas flow rate, gas nozzle size, and gas nozzle posture on the protection effect were analyzed systematically by experiments and the optimum gas nozzle posture was obtained.

## 2. Materials and Methods

### 2.1. Materials and Experimental Procedures

**Figure 1:**
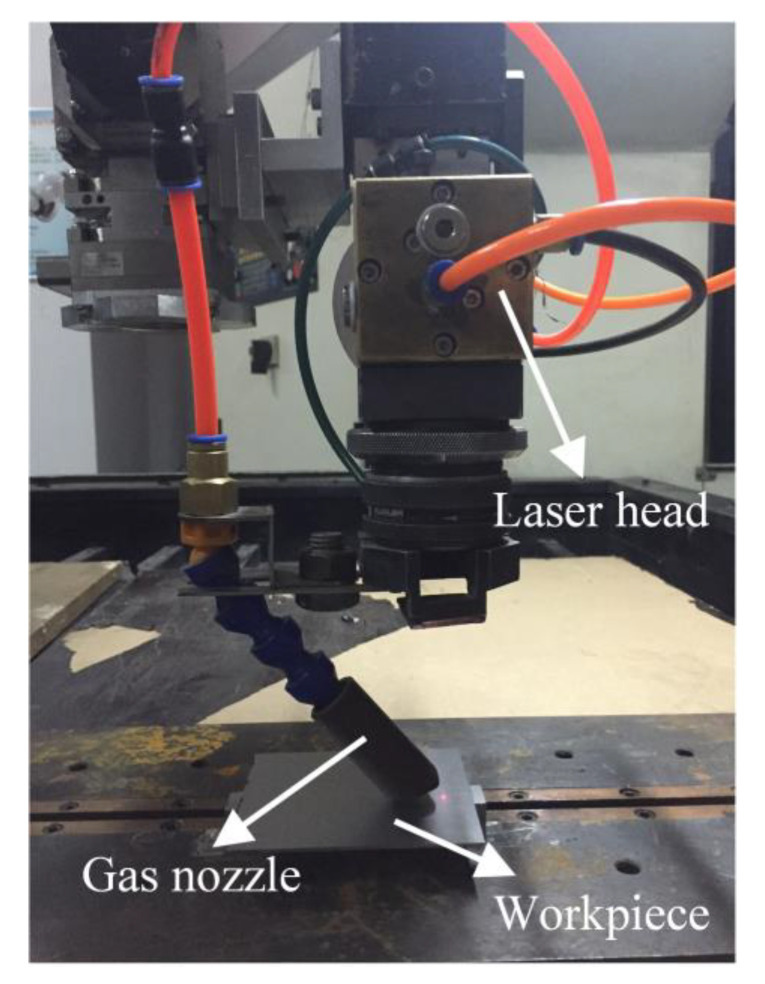
Laser processing system used in the experiment.

The as-rolled TC4 with dimensions of 80 mm × 60 mm × 1.6 mm and the as-cast TC4 with dimensions of 100 mm × 15 mm × 15 mm were selected as the upper plate and bottom plate, respectively. The chemical composition of TC4 used in this investigation is shown in Table 1. The chemical composition was detected using an inductively coupled plasma spectrometer (ICP) in the Analysis Test and Computing Center of Harbin Institute of Technology. Figure 1 shows the diffusion-cooled CO_2_ laser processing system used in the experiment. The maximum laser power is 3 kW, the wavelength is 10.6 μm, the focal distance is 200 mm, and the focal spot diameter is 0.2 mm.

The base metals were cleaned with acetone before laser welding. Argon was employed via a paraxial nozzle as the shielding gas. Laser power (*P*) kept 1800 W constantly, and welding speed (*v*) varied to change the heat input. The process was observed by an industrial high-speed CCD. Thermocouples were spot welded on the base metal surface to measure the temperatures. Visualization of the shielding gas flow was realized by a Schlieren system [28].

The metallographic samples were ground by abrasive papers, polished with diamond compounds, and then etched by mixed acid solution (1 vol.% HF + 1.5 vol.% HCl + 2.5 vol.% HNO_3_ + 95 vol.% H_2_O). An optical microscope (OM) and scanning electron microscope (SEM) were employed to observe the cross-section morphology of the welds. As for weld dimensions, *B* represents the width of welds, and *H* represents penetration depth. An HVS-1000 Z microhardness tester was used to examine Vickers hardness, and 200 g were loaded to each spot for 10 s. The indentations were spaced by 200 μm.

### 2.2. Calculation of Temperature Field of Laser Welding

**Figure 2:**
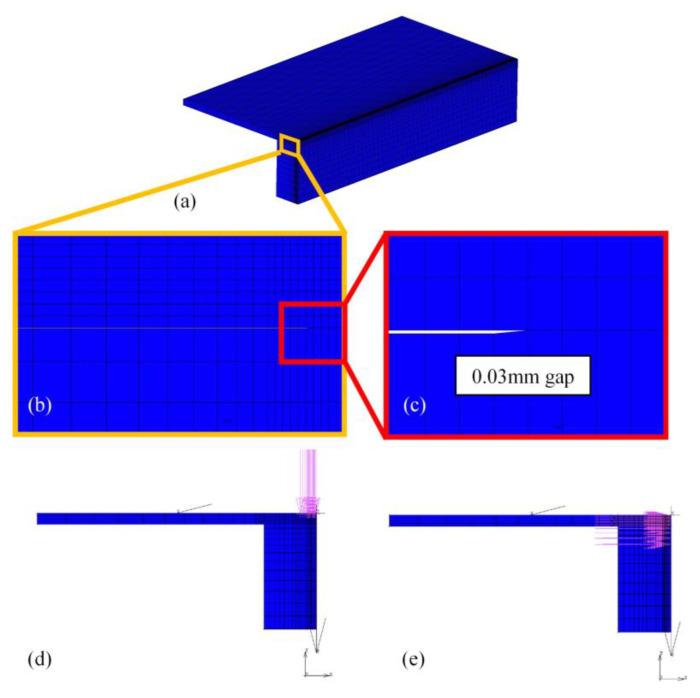
Mesh model of the temperature field: (**a**) mesh model, (**b**) mesh transition, (**c**) gap between plates, (**d**) action domain of Gaussian surface heat source, and (**e**) action domain of conical heat source.

Due to the symmetry of the workpieces, models were set as Figure 2. Meshes in the weld were fine and sparse in base metal with smooth transition. To enhance the accuracy, a 0.03 mm gap was set between the two plates where they were not connected. In this investigation, the heat source was separated into two parts: a Gaussian surface heat source simplified from plasma and a conical heat source from a keyhole. The domain of action of the two heat sources is shown in Figure 2d,e. The heat flux distribution of the surface heat source was:

(1) $$q_{s}=\frac{\alpha Q_{s}}{\pi r_{s}^{2}}exp\left[-\frac{\alpha \left(x^{2}+y^{2}\right)}{r_{s}^{2}}\right],$$

where α is the heat flux concentration coefficient, *Q_s_* is the power of the surface heat source, and *r_s_* is the effective radius of the surface heat source. The volumetric hear source could be described as:

(2) $$q_{v}\left(x,y\right)=\frac{6Q_{v}}{\left(2\pi r_{v}^{2}h+\beta \pi r_{v}h^{2}\right)}exp\left[\frac{-3\left(x^{2}+y^{2}\right)}{r_{v}^{2}}\right]\frac{\left(\beta z+r_{v}\right)}{r_{v}},$$

where *Q_v_* is the power of the volumetric heat source, *r_v_* is the effective radius of the volumetric heat source, *h* is the effective height of the volumetric heat source, and *β* is the energy attenuation coefficient of the volumetric heat source. The correlation between *Q_s_* and *Q_v_* is:

(3) $$\eta P=Q_{s}+Q_{v},$$

where *η* is the efficiency, and *P* is the laser beam power. A subroutine realized that the surface heat source was employed for surface elements and the volumetric heat source was implemented for body elements, respectively.

The boundary condition in this investigation is the heat transfer between the plate surfaces and the atmosphere. As a result of the shielding gas flow, the convection above the weld bead was stronger. Owing to the relatively low temperature, the heat exchange coefficient of the bottom surface of the bottom plate was considered to be the same as other surfaces. Additionally, there was no heat exchange on the symmetric plane.

### 2.3. Model of Shielding Gas Behavior

**Figure 3:**
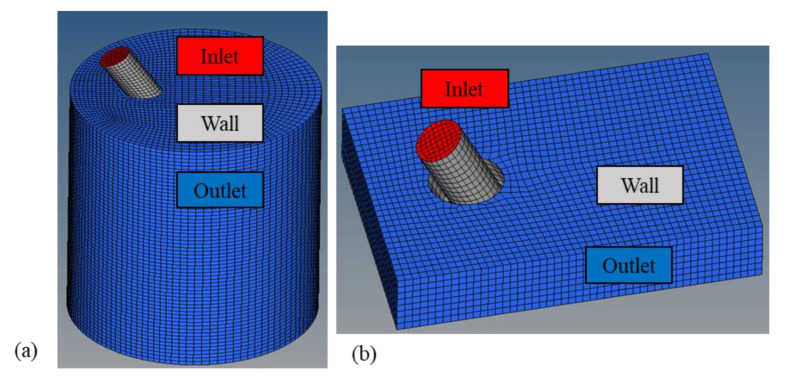
Mesh models of calculated gas field: (**a**) unrestrained and (**b**) restrained.

The modeling of the shielding gas behavior was divided into two parts: unrestrained and restrained conditions. For unrestrained conditions, the computational field was set as a cylinder 150 mm high and 120 mm in diameter. As for the restrained condition, a cuboid with dimensions of 120 mm × 80 mm × 10 mm was defined as the field. Furthermore, 10 mm represent the distance between the nozzle and the plate. Figure 3 demonstrates the geometry and mesh model of the calculated field. Red, blue, and white regions were the inlets, outlets, and walls, respectively. In the initial condition, static air at 101,325 Pa filled the computational field.

The standard *k*-*ε* turbulence model [8] was applied in the simulation, and the model could be described as:

(4) $$\frac{\partial }{\partial }\left(\rho k\right)+\frac{\partial }{\partial x_{i}}\left(\rho ku_{i}\right)=\frac{\partial }{\partial x_{i}}\left[\left(\mu +\frac{\mu_{t}}{\sigma_{k}}\right)\frac{\partial k}{\partial x_{i}}\right]+P_{k}+P_{b}-\rho \varepsilon ,$$

(5) $$\frac{\partial }{\partial }\left(\rho \varepsilon \right)+\frac{\partial }{\partial x_{i}}\left(\rho \varepsilon u_{i}\right)=\frac{\partial }{\partial x_{i}}\left[\left(\mu +\frac{\mu_{t}}{\sigma_{\varepsilon }}\right)\frac{\partial \varepsilon }{\partial x_{i}}\right]+C_{1}\frac{\varepsilon }{k}\left({{P_{k}+C}_{3}P}_{b}\right)-C_{2}\rho \frac{\varepsilon^{2}}{k},$$

(6) $$\mu_{t}={\rho C}_{\mu }\frac{k^{2}}{\varepsilon },$$

(7) $$P_{k}=\mu_{t}S^{2}$$

(8) $$P_{b}=-\frac{g_{i}\mu_{t}}{\rho {Pr}_{t}}\frac{\partial T}{\partial x_{i}}{\left(\frac{\partial \rho }{\partial T}\right)}_{p},$$

where all the parameters reported by Tani et al. [29] are listed in Table 2. Additionally, constants *C*_1_, *C*_2_, *C*_3_, *C_μ_*, *σ_k_*, *σ_ε_*, and *Pr_t_* took the default values from the software Fluent 16.0. Heat input and plasma were ignored in the simulation of gas behavior.

## 3. Results and Discussions

### 3.1. Model Validation

**Figure 4:**
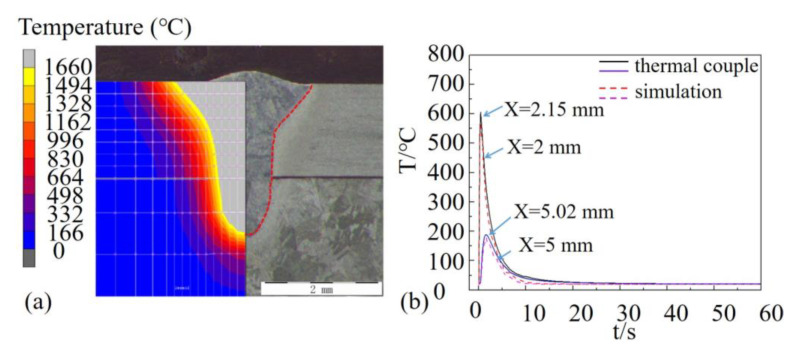
Validation of temperature field model: (**a**) comparison of simulated and experimentally acquired cross-sections and (**b**) comparison of simulated and experimentally acquired temperature–time curves.

Experiments were carried out at a welding speed of 1.8 m/min to validate the numerical model. The simulation of the temperature field is presented in Figure 4. Figure 4a shows the keyhole and molten pool explicitly. The accurate match of temperature distribution and weld profile of the cross-section, which was a distinct nail shape, primarily verified the simulation. In addition, temperatures of specific positions on the surface of the plates were reconfirmed. Temperatures of spots 2 mm and 5 mm from the weld center perpendicular to the welding direction were detected by thermocouples. Node temperatures of 2.15 mm and 5.02 mm from the weld center were also extracted from the simulation. The acceptable discrepancies between the temperature–time curves revealed accurate modeling of the heat sources and precise boundary conditions.

**Figure 5:**
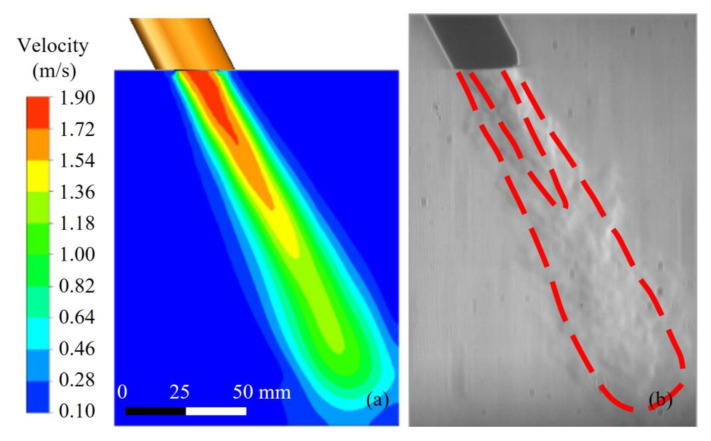
Validation of shielding gas flow under unrestrained conditions: (**a**) velocity distribution and (**b**) Schlieren image.

**Figure 6:**
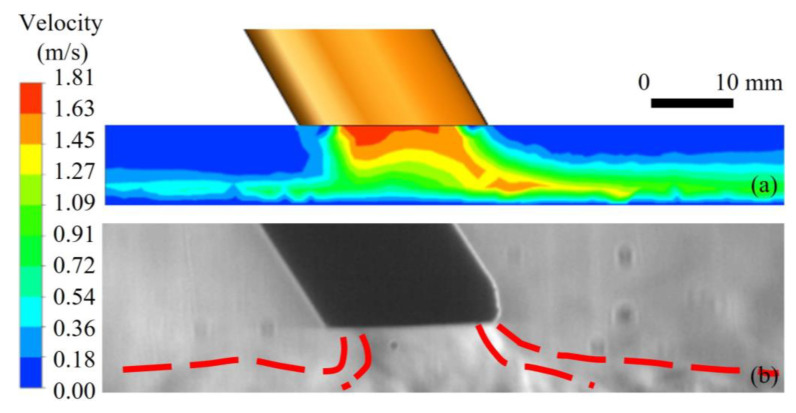
Validation of shielding gas flow under restrained conditions: (**a**) velocity distribution and (**b**) Schlieren image.

Figure 5 and Figure 6 show the comparison of gas flow velocity distribution and images obtained by the Schlieren method under unrestrained and restrained conditions. Red dashed lines represented the flow boundaries of shielding gas. The velocity field distribution of shielding gas outside the nozzle reflects the gas flow ranges and flow gradient, as shown in Figure 5a and Figure 6a. The Schlieren images reflect the sparsity and flow characteristics of the shielding gas. By comparing the simulation results with the Schlieren images in Figure 5b and Figure 6b, it is found that the velocity field distribution is consistent with the real gas flow situations. The consistency of the numerical simulation results with actual Schlieren images showed that the model established for this experiment was able to truly represent the flow of shielding gas in the air from the nozzle.

### 3.2. Threshold Temperature of Gas Protection

**Figure 7:**
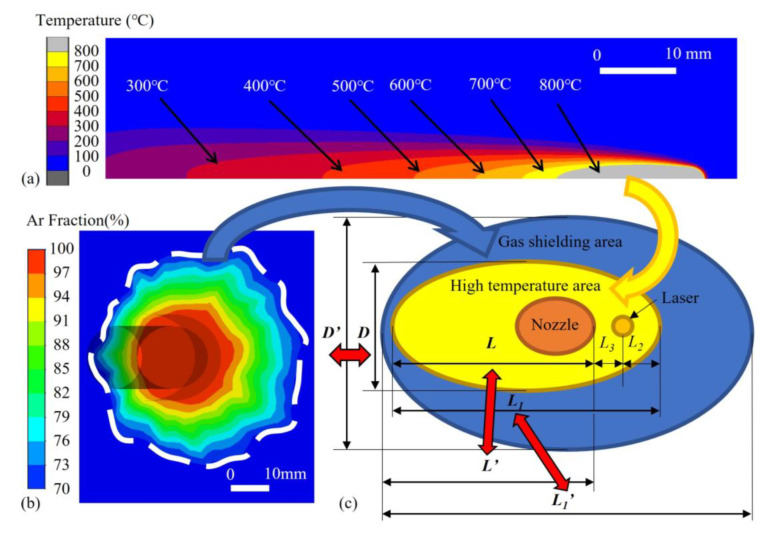
Typical results of simulations: (**a**) high-temperature area, (**b**) gas shielded area, and (**c**) schematic of the parameters studied in the experiment.

Figure 7 shows the typical simulations of the analyzed area on the plate surface and their combination. High-temperature areas showed an ellipse shape, and the 70% area argon took up was labeled as the gas shielding area. The two areas were matched with the positions of the nozzle and laser. The definitions of analyzed area dimensions and the investigated gas parameters are listed in detail in Table 3. According to the results of the aforementioned temperature and gas flow field, the dimensions of the area above a certain temperature and under given gas parameters could be obtained. As long as *D*′ > *D*, *L*_1′_ > *L*_1_, *L*′ > *L*, the area above a certain temperature was considered to be covered by the gas flow.

**Figure 8:**
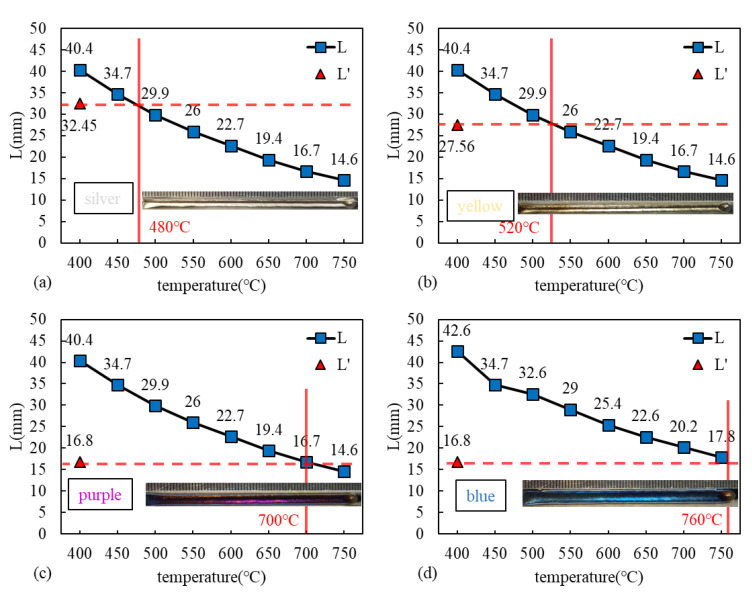
*L* and *L′* under various welding parameters listed in Table 4 and the corresponding weld surface color: (**a**) case a, (**b**) case b, (**c**) case c, and (**d**) case d.

*L* and *L′* under various welding parameters are listed in Table 4, and the corresponding weld surface color is shown in Figure 8. In each case, *L′* was obtained first under the investigated gas parameters and was compared with the temperature of the protected area. Then, the relationship between the temperature of the protected area and the color of the weld was analyzed. When *L′* was 32.45 mm (case a) and the lowest temperature of the gas-shielded area was 480 °C, the weld surface turned out to be silver. With the reduction in the nozzle’s inner diameter, the gas-shielded area decreased. *L′*, in case b and c, was 27.56 mm and 16.8 mm, respectively. The corresponding lowest temperature of the shielded area was 520 °C and 700 °C, respectively, and the weld surface color changed from silver to yellow and purple. In case d, *L′* was kept at 16.8 mm, and the lowest temperature of the shielded area was increased to 760 °C by increasing the heat input (decreasing the welding speed from 1.8 m/min to 1.2 m/min). The weld surface turned out to be blue, indicating a severe oxidation degree. Since only silver weld could be considered as well protected, the threshold protection temperature of titanium laser beam welding was determined to be 480 °C.

### 3.3. Influence of Shielding Gas Parameters on Gas Protection Effect

#### 3.3.1. Influence of Gas Flow Rate

**Figure 9:**
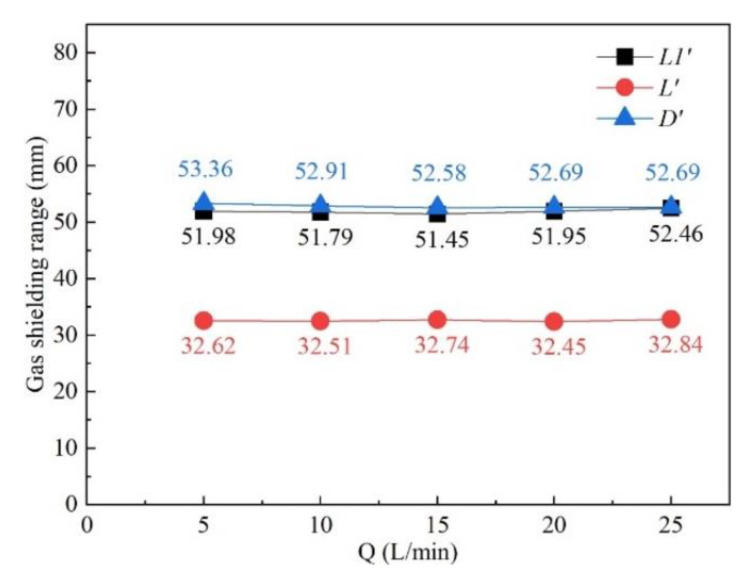
Influence of gas flow rate on gas shielding area dimensions.

**Figure 10:**
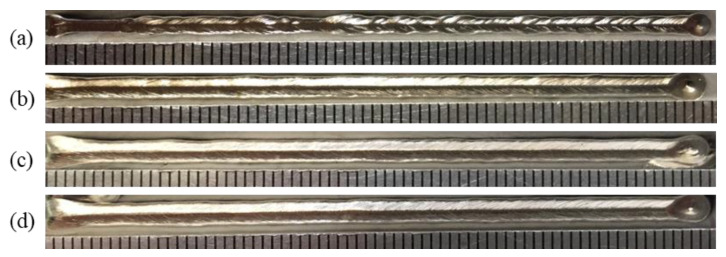
Weld appearances under different gas flow rates: (**a**) 10 L/min, (**b**) 15 L/min, (**c**) 20 L/min, and (**d**) 25 L/min.

The influence of gas flow rate on the shielded range was investigated with gas flow rates of 10 L/min, 15 L/min, 20 L/min, and 25 L/min, while other process parameters were fixed as *d* = 16 mm, *θ* = 60°, *h* = 5 mm, and *v* = 1.8 m/min. Figure 9 shows the influence of gas flow rate on gas shielding area dimensions. It can be seen from Figure 9 that the gas shielding area dimensions under various gas flow rates exhibited a stable trend. Thus, the gas flow rate played a negligible role in the protective range. Compared with the 480 °C area (*L* = 32.00 mm, *D* = 5.60 mm), all the weld beads were well protected, which was verified by the silver weld surfaces in Figure 10. However, only considering the shielded range was not sufficient to describe the gas protection effect. For instance, in Figure 10a, sags and crests were seen on the weld surface, because the gas flow rate of 10 L/min was too small to restrain the plasma plume and stabilize the laser welding process. When the gas flow was small, the gas moved exclusively by its own diffusion, reaching the surface of the workpiece at a low velocity, which resulted in poor weld surface gloss. Meanwhile, larger gas pressure on the surface of the workpiece contributed to the inhibition of plume plasma. In consequence, shielding gas pressure at different points on the upper surface of the workpiece along the center line of the weld and gas velocity at various points from the gas nozzle at 2 mm from the top surface of the workpiece were investigated to obtain a better gas protection effect.

**Figure 11:**
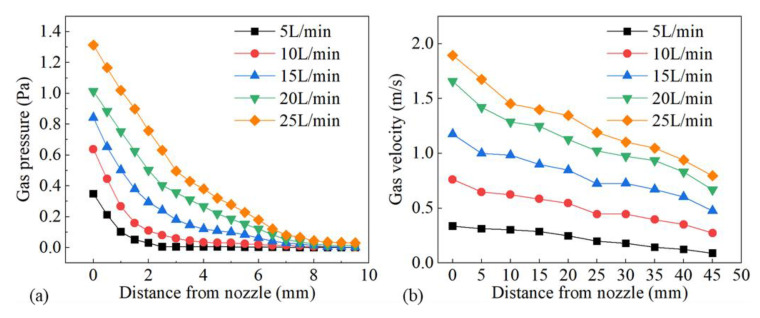
Influence of gas flow rate on (**a**) gas pressure and (**b**) gas velocity.

**Figure 12:**
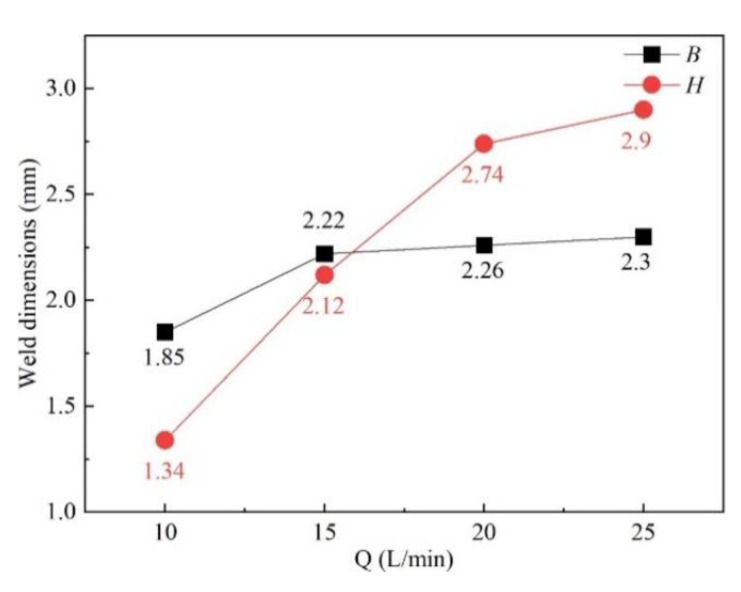
Influence of gas flow rate on weld dimensions.

**Figure 13:**
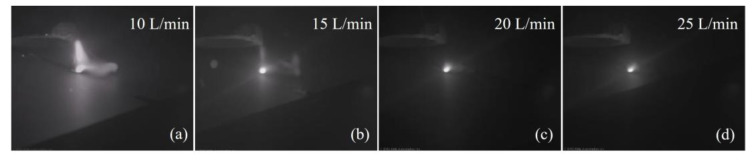
Plasma plume morphologies under different gas flow rates: (**a**) 10 L/min, (**b**) 15 L/min, (**c**) 20 L/min, and (**d**) 25 L/min.

Figure 11 shows the influence of gas flow rate on gas pressure and gas velocity. As the gas flow rate increased, both gas pressure and gas velocity went up, as shown in Figure 11a,b, which led to better restrain and less energy absorption of the plasma plume. It was confirmed by the increasing weld width and penetration statistically demonstrated in Figure 12. Figure 13 shows the plasma plume morphologies under different gas flow rates using high-speed photography. High-speed photography images showed that the dimensions of the plasma plume shortened with the increase in gas flow rate.

#### 3.3.2. Influence of Nozzle Height

**Figure 14:**
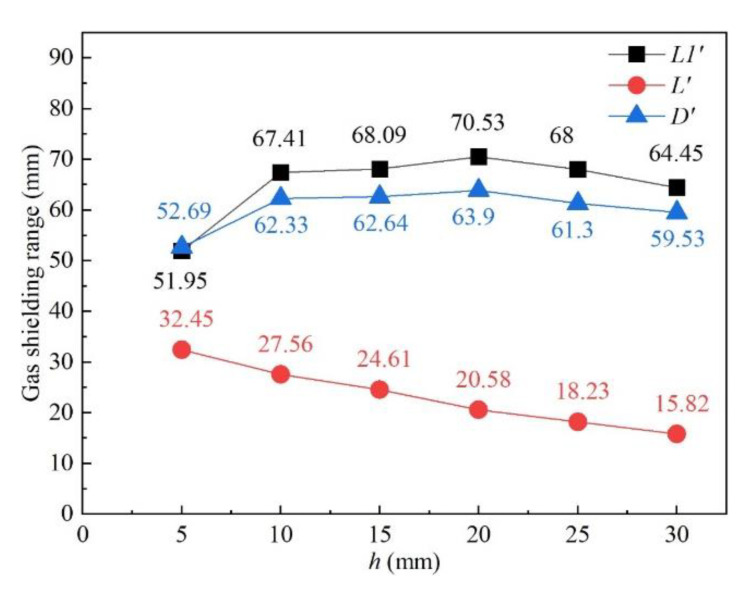
Influence of nozzle height on gas shielding area dimensions.

**Figure 15:**
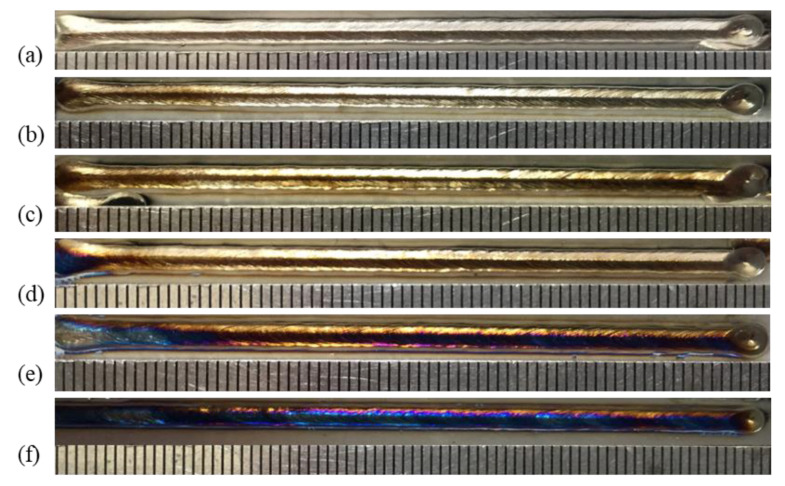
Weld appearances under different nozzle heights: (**a**) 5 mm, (**b**) 10 mm, (**c**) 15 mm, (**d**) 20 mm, (**e**) 25 mm, and (**f**) 30 mm.

**Figure 16:**
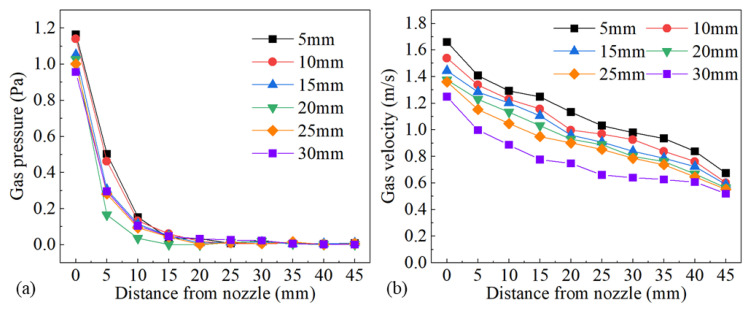
Influence of nozzle height on (**a**) gas pressure and (**b**) gas velocity.

**Figure 17:**
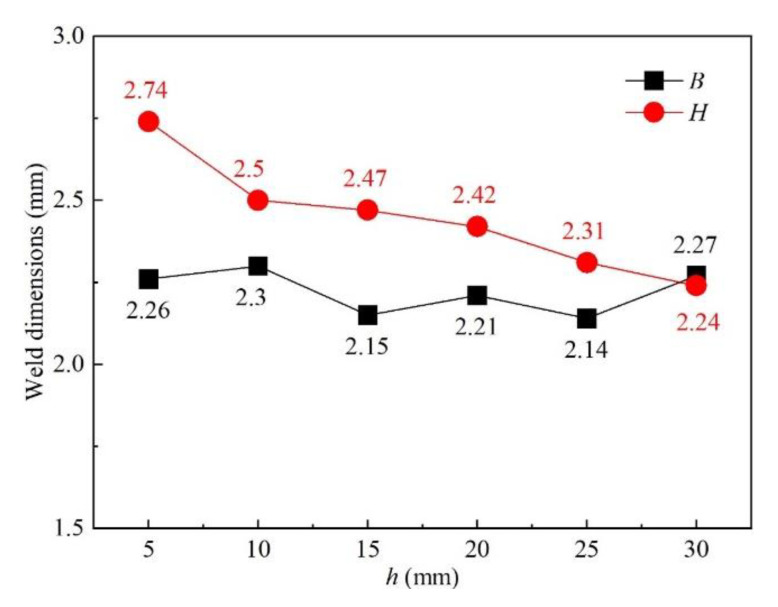
The influence of nozzle height on weld dimensions.

The influence of nozzle height from the workpiece surface on shielding area dimensions was investigated under nozzle heights of 5 mm, 10 mm, 15 mm, 20 mm, 25 mm, and 30 mm, while other process parameters were fixed as *d* = 16 mm, *θ* = 60°, *Q* = 20 L/min, *P* = 1800 W, and *v* = 1.8 m/min. Figure 14 shows the influence of nozzle height on gas shielding area dimensions. When *h* increased, *L′* decreased permanently while *L*1′ and *D′* increased firstly and then decreased. When *h* is too small, gas rebounded by the plate may perturb the newly blown gas, which explains the rise in *L1′* and *D′*; when *h* was too large, gas flow turned turbulent on the plate, and air could be involved more easily, which resulted in the drop. The calculated results of the high-temperature area of 480 °C were *L* = 32.00 mm and *D* = 5.60 mm. When the nozzle height was 5 mm, *L′* was 32.45 mm, which was greater than *L*. As the nozzle height increased, *L′* reduced to below 32 mm from 27.56 mm at 10 mm nozzle height to 15.82 mm at 30 mm nozzle height. Comparing *L′* with *L*, the welding process could not be well protected when *h* was larger than 5 mm, which was validated by the experiments in Figure 15. Figure 15 shows the weld appearances under different nozzle heights. The weld was soundly protected when the nozzle height was 5 mm. The weld appearances deteriorated with the increase in nozzle height, presented by worse and worse oxidation. The surface turned purple and blue when *h* was 30 mm. With the increase in nozzle height, the gas pressure and velocity of shielding gas decreased, as shown in Figure 16, which led to the decline of restrain of the plasma. The pressure was from spots on the plate surface along the weld center with different distances from the nozzle outlet, and the velocity was from spots 2 mm above the surface. The decline led to greater plasma and lower laser energy efficiency, which is in accordance with the weld penetration decrease in Figure 17.

#### 3.3.3. Influence of Nozzle Inner Diameter

**Figure 18:**
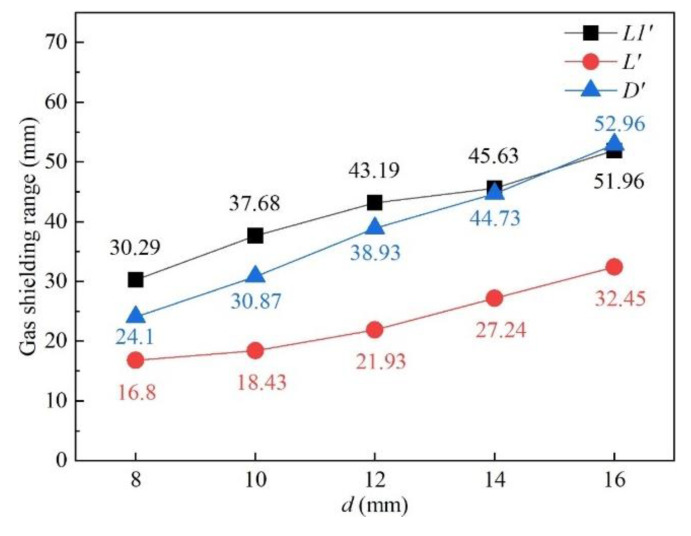
Influence of nozzle inner diameter on gas shielding area dimensions.

**Figure 19:**
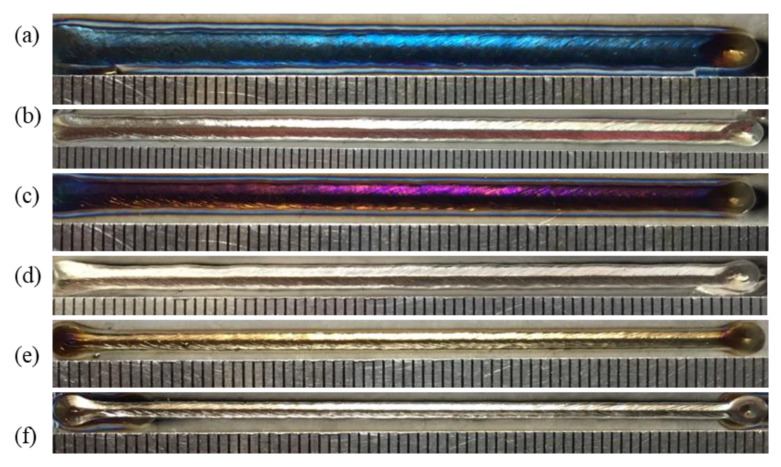
Weld appearances under different nozzle inner diameters: (**a**,**c**,**e**) 8 mm and (**b**,**d**,**f**) 16 mm.

**Figure 20:**
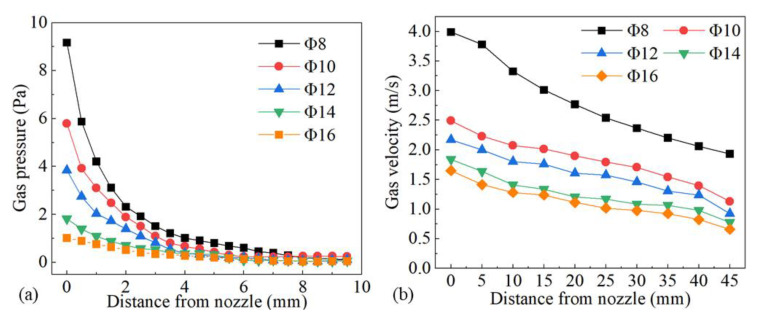
Influence of nozzle inner diameter on (**a**) gas pressure and (**b**) gas velocity.

**Figure 21:**
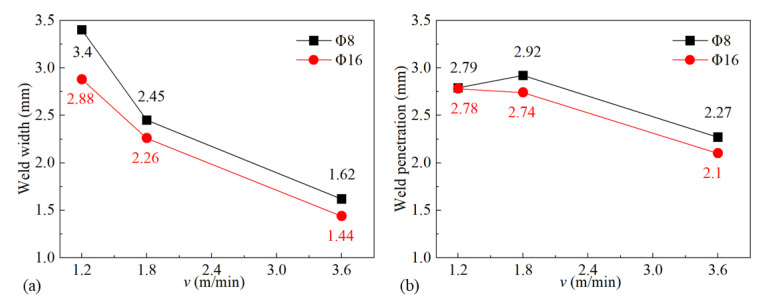
Influence of nozzle inner diameter on weld dimensions under different welding speeds: (**a**) weld width and (**b**) weld penetration.

Simulations were implemented to investigate the influence of 8 mm, 10 mm, 12 mm, 14 mm, and 16 mm nozzle inner diameter on the gas shielded range and other gas parameters were kept to *h* = 5 mm, *θ* = 60°, and *Q* = 20 L/min. Figure 18 shows the influence of the nozzle’s inner diameter on gas shielding area dimensions. It could be observed that *L′*, *L*1′, and *D′* were positively associated with nozzle inner diameter *d*. Compared with the calculated high-temperature area of 480 °C (*L* = 32.00 mm, *D* = 5.60 mm), only gas flow from the 16 mm nozzle could shield the welding process effectively. Experiments were carried out according to the parameters listed in Table 5. Figure 19 shows weld appearances under different nozzle inner diameters. Compared with a 16 mm diameter gas nozzle, the application range of an 8 mm nozzle is less, and it could hardly protect the welding process with relatively high heat input (Figure 19a,c,e), while a 16 mm diameter gas nozzle exhibited a great effect on the weld protection (Figure 19b,d,f). Figure 20 shows the influence of the nozzle’s inner diameter on gas pressure and gas velocity. With the increase in the nozzle’s inner diameter, the pressure and velocity of shielding gas on the surface of the plate fell, which meant enlarging the nozzle’s inner diameter was bad for the suppression of plasma. Figure 21 shows the influence of nozzle inner diameter on weld width and weld penetration. Larger weld penetration and width using the 8 mm nozzle illustrated that with the increase in gas nozzle diameter, the suppression degree of the plasma plume effect was reduced.

#### 3.3.4. Influence of Nozzle Angle

**Figure 22:**
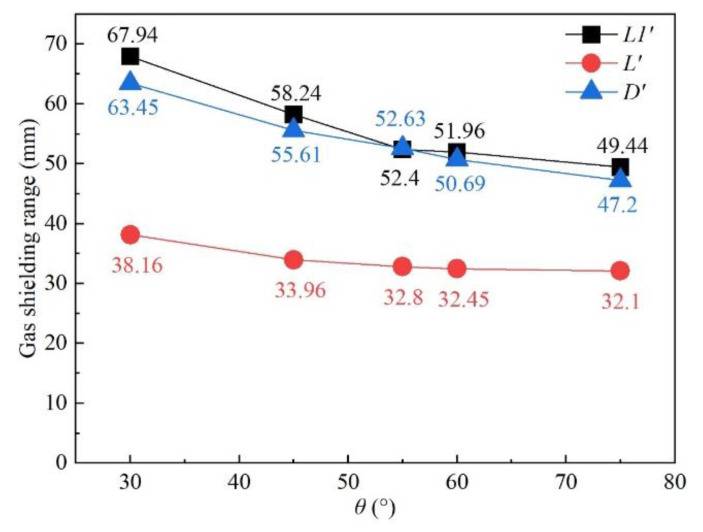
Influence of nozzle angle on gas shielding area dimensions.

**Figure 23:**
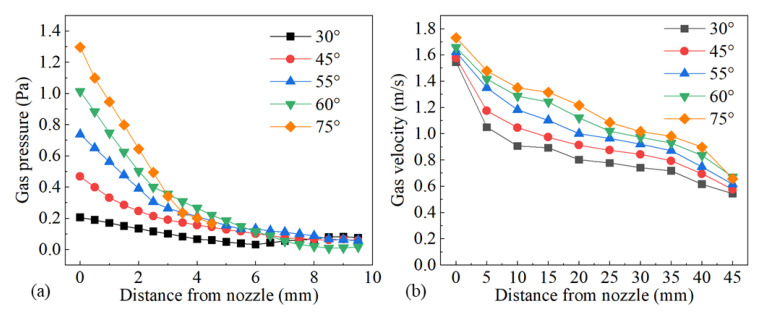
Influence of nozzle angle on (**a**) gas pressure and (**b**) gas velocity.

The influence of nozzle angle on shielded range was studied under nozzle angles of 30°, 45°, 55°, 60°, and 75°, while other process parameters were fixed as *d* = 16 mm and *h* = 5 mm. The nozzle angle was defined as the angle between the gas nozzle and the workpiece surface. Figure 22 shows the influence of the nozzle angle on gas shielding area dimensions. When the nozzle angle rose from 30° to 55°, *L′*, *L*1′, and *D′* dropped off distinctly, and when the angle varied from 55° to 75°, the shielded range hardly changed. Compared with the calculated high-temperature area of 480 °C (*L* = 32.00 mm, *D* = 5.60 mm), the welding process could all be well protected with a nozzle angle between 30° and 75°. Figure 23 shows the influence of nozzle angle on gas pressure and gas velocity. The increase in nozzle angle enhanced the pressure and velocity of shielding gas on the surface of the workpiece surface. It could be inferred that increasing the nozzle angle was beneficial to the restraining of the plasma.

#### 3.3.5. Microstructure and Microhardness of Welds

**Figure 24:**
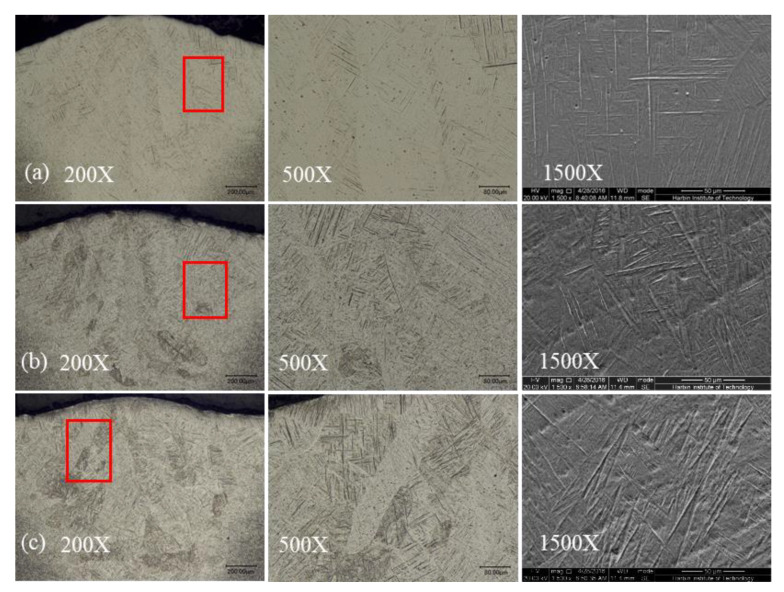
Microstructures of weld zones with different surface colors: (**a**) silver, (**b**) yellow, and (**c**) bluish violet.

Different weld surface colors could be obtained by various protection in previous experiments. When nozzle height *h* varied from 5 mm to 30 mm, weld surfaces appeared silver, yellow, and bluish violet, respectively (*v* = 1.8 m/min). Figure 24 shows the OM images of the weld cross-section microstructure with different weld surface colors. It revealed that acicular martensite is distributed as a parallel bundle or basket and weaved in columnar β grains. When protection was good enough, the weld turned out to silver, and fine martensite was parallel and acicular in the fusion zone, as shown in Figure 24a. When shielding was poor, more and larger martensites appeared densely, with basket and weave distribution (Figure 24b,c).

**Figure 25:**
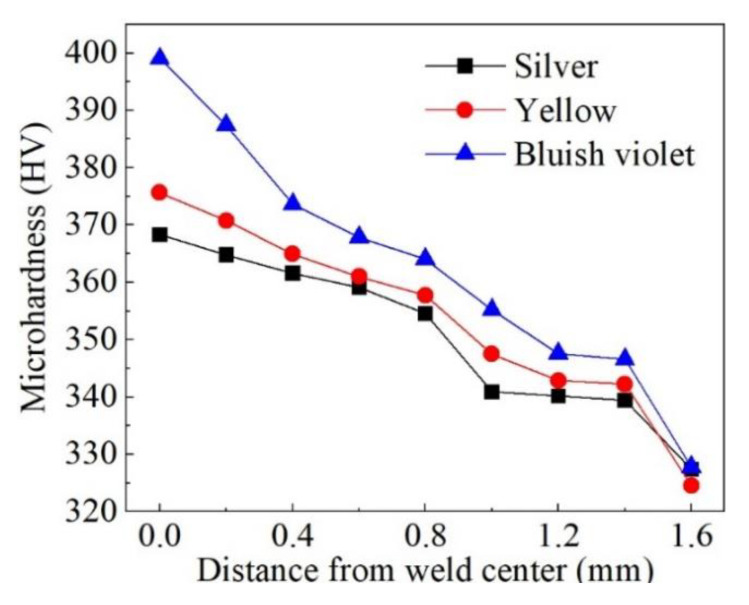
Microhardness of weld zone with different surface colors.

Figure 25 shows the variation in microhardness of welds with different weld surface colors from weld center to base metal. The average microhardness of base metal was 327 HV, while that of silver weld, yellow weld, and bluish violet weld was 363.7 HV, 367.0 HV, and 382.1 HV. When the process was not shielded adequately, oxygen and nitrogen entered the melting pool and formed a solid solution, which played the role of inhibiting the dislocation movement attributed to lattice distortion. Eventually, the microhardness of the weld was enhanced [30,31].

This explained that the microhardness of the silver weld was the lowest and that of the bluish-violet weld was the highest. Meanwhile, nitrogen and oxygen promoted the transformation of β columnar grains in the weld center to the needle-like α’ phase, resulting in an increase in phase boundaries and a high density of twins and dislocations in the α’ phase [32], which contributed to the increment of weld hardness.

## 4. Conclusions

In this work, models of both temperature field and shielding gas behavior were set up and validated, and the influences of gas parameters on the gas protection effect were studied by experiments. The main conclusions are as follows:

(1) The results of temperature and gas velocity distribution obtained by simulation were both well matched with experiments, so the numerical model established in this work was validated. The threshold gas protection temperature of TC4 alloy in the laser welding process was found to be 480 °C by combining the simulation and experiment results.
(2) The nozzle height, nozzle inner diameter, and nozzle angle principally influenced the gas protection effect of TC4 alloy in the laser welding process. Lowering the nozzle height properly, enlarging the nozzle’s inner diameter, and decreasing the nozzle angle contributed to a better gas protection effect.
(3) Gas flow rate and nozzle inner diameter mainly influenced the restraining of the plasma plume. Increasing the gas flow rate and shrinking the nozzle inner diameter benefited the restraining of the plasma plume and enhanced the laser energy utilization rate.

## Figures and Tables

**Table 1:** Chemical composition of TC4 (wt.%).

Al	V	Fe	Si	C	N	H	O	Ti
5.96	3.74	0.096	0.03	0.01	0.013	0.0048	0.05	Bal.

**Table 2:** The model variables, constants, and parameters used in CFD analysis.

Model Variables	Model Constants	Model Parameters
*k*, turbulent kinetic energy	*T*, temperature	*C*_1_ = 1.44
*ε*, turbulent dissipation	*ρ*, density of shielding gas	*C*_2_ = 1.92
*t*, time	*μ*, viscosity of shielding gas	*C*_3_ = 0.09
*x_i_*, space coordinates in the *i*th direction	*S*, modulus of mean rate-of-strain tensor	*C_μ_* = 0.09
*u_i_*, component of the velocity vector in the *i*th direction	*g_i_*, component of the gravitational vector in the *i*th direction	*σ_k_* = 1.0
—	—	*σ_ε_* = 1.3
		*Pr_t_* = 0.85

**Table 3:** The definitions of analyzed area dimensions and the investigated gas parameters.

Variables	Definitions
*D*	Width of high-temperature area
*L*_1_	Length of high-temperature area
*L*	Distance between the projection of nozzle tip and the end of the high-temperature area
*D′*	Width of gas shielding area
*L*_1′_	Length of gas shielding area
*L′*	Distance between the projection of the nozzle tip and the end of the gas shielding area
*d*	Inner diameter of the nozzle
*Q*	Gas flow rate
*θ*	Angle between the axle of the nozzle and the surface of the plate
*h*	Distance between the nozzle tip and the surface of the plate

**Table 4:** Laser welding parameters of the experiments depicted in Figure 8.

Case	Welding Speed (m/min)	Nozzle Height (mm)	Nozzle Inner Diameter (mm)
a	1.8	5	16
b	1.8	5	12
c	1.8	5	8
d	1.2	5	8

**Table 5:** Process parameters used under different nozzle inner diameters and welding speeds.

Case	Nozzle Inner Diameter (mm)	Welding Speed (m/min)
a	8	1.2
b	16	1.2
c	8	1.8
d	16	1.8
e	8	3.6
f	16	3.6

## Data Availability

The data used to support the findings of this study are available from the corresponding author upon request.
